# Advantages and Limitations in the Evaluation of the Neurological and Functional Deficit in Patients with Spinal Cord Injuries

**DOI:** 10.3390/clinpract13010002

**Published:** 2022-12-27

**Authors:** Camelia Florentina Lascu, Camelia Liana Buhaș, Gabriel Mihai Mekeres, Mădălin Bulzan, Robert Bogdan Boț, Georgiana Albina Căiță, Ioan Bogdan Voiță, Mihaela Dana Pogan

**Affiliations:** 1 Doctoral School of Biomedical Sciences, Faculty of Medicine and Pharmacy, University of Oradea, 410087 Oradea, Romania; 2 Morphological Disciplines Department, Faculty of Medicine and Pharmacy, University of Oradea, 410087 Oradea, Romania; 3 Department of Legal Medicine, County Clinical Emergency Hospital of Oradea, 410169 Oradea, Romania; 4 Department of Anesthesiology and Intensive Care, Regional Institute of Gastroenterology and Hepatology “Prof. Octavian Fodor”, 400162 Cluj-Napoca, Romania; 5 Department of Dental Medicine, Faculty of Medicine and Pharmacy, University of Oradea, 410087 Oradea, Romania

**Keywords:** neurological deficit, spinal cord injury, evaluation scales, functional prognosis

## Abstract

(1) Background: Vertebro-medullary trauma (VMT) causes osteo-articular injuries in a varied anatomical lesion associated with multiple clinical manifestations and therapeutic indications. The neurological evaluation of patients who have suffered a spinal cord injury (SCI) is costly in testing the motor and sensory function. To standardize the assessment, several scales are used that measure the neurological deficit in order to guide subsequent treatment according to complete or incomplete SCI. The aim of this study is to identify and present the relevant tools for assessing SCI. (2) Methods: Relevant SCI studies were used for a fact-finding investigation from a rational and critical perspective of this field of research. The relationship between clinical tools and those with a psychosocial component was assessed based on studies reported in the literature. (3) Results: SCI severity scales have been proposed throughout to be able to estimate the functional prognosis of victims of these traumatic events. These tools can be divided into scales for assessing the neurological deficit due to trauma, and functional scales that assess the ability to perform daily activities, self-care, etc. (4) Conclusions: The closest scale to the need for standardization and the most accurate assessment of neurological deficits secondary to SCI is ASIA/IMSOP.

## 1. Introduction

Vertebro-medullary trauma (VMT) causes osteo-articular injuries of the vertebrae and their contents (marrow, nerve roots, meninges and vessels) in a varied anatomical lesional association, with multiple clinical manifestations and therapeutic indications [1]. The occurrence of a spinal cord injury (SCI) causes a disability that can manifest itself in different levels of severity, the patient having difficulties in the family, social and economic context of functioning and integration [2,3]. SCI patients face poor financial situations and poor socio-economic achievements, and the life expectancy of the disabled person is much lower [4,5]. Worldwide, there has been an obvious concern for the development and implementation of policies and programs to improve the quality of life of people with disabilities [6,7]. The aim is to ensure people’s access to specific medical services, education and viable employment opportunities [8,9,10]. It is important to add that SCI mainly affects the active population, the average age being 35 years, so the economic impact is great [11,12,13]. Another worrying aspect is the increasing incidence of patients with complete spinal cord section and quadriplegia, which implies increased care needs [14,15,16].

In order to determine the neurological and functional deficit in patients with vertebral-medullary injuries, different evaluation scales were developed. These scales have been validated and improved over the years to determine a predictive tool for the functional outcomes of patients with SCI [17,18].

Our objective is to analyze the advantages and limitations of the current scales for evaluating the neurological and functional deficit in patients with SCI.

## 2. Materials and Methods

**Figure 1:**
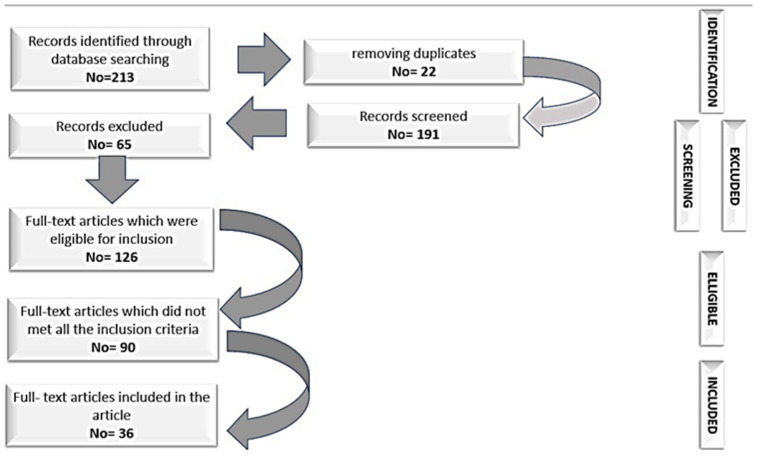
The selection process.

We present the tools that have proven to be a reliable standard and have direct utility in the work of clinicians. Studies supporting the fidelity and validity of SCI research and assessment tools will be presented according to the instrument presented. Relevant SCI studies were used for a fact-finding investigation from a rational and critical perspective of this field of research. The relationship between clinical tools and those with a psychosocial component was assessed based on studies reported in the literature. To achieve this goal, SCI severity and functional prognosis scales were analyzed using Medline, PubMed, Scopus, Proquest, Science Direct, Springerlink, and WOS bases, including relevant keywords supported by internationally established sites in the field, or regionally (such as the International Spinal Cord Society). In order to select the articles, we utilized the following keywords: scales, spinal cord injury, vertebro-medullary trauma, neurologic recovery, functional recovery, neurologic deficit. Studies evaluating SCI in children were excluded due to the multitude of factors that would have distorted the presented information. The study will reveal the screening criteria for inclusion and the exclusion of studies. First of all, we selected from international journals the studies that validated these scales and which presented as relevant sources of empirical and meta-analytical data. Second, to avoid misinterpretation, the selected works included only articles published in English. Thirdly, in terms of chronology, a period of 20 years was selected. The selection of studies has been implemented to ensure sufficient time to observe the evolution of international research on SCI. We believe that we offer a sufficiently long time-frame to be able to identify the elements relevant to our SCI investigation. The eligibility of studies with relevant statistical data was the last step in which an additional and more in-depth examination of the literature was performed. Consequently, this step was aimed at reviewing the titles, abstracts, and main content of each type of study, research article, validation study, and quantitative meta-analysis to ensure that they meet the inclusion criteria. The selection process for this article is illustrated in Figure 1.

## 3. Results and Discussion

The need for standardization and accurate assessment of neurological deficits secondary to SCI has led to the development of various scales for their quantification, but none of them can be considered ideal, as each has its advantages and disadvantages. The choice of one or another of the scales also depends on the preferences of the doctor who uses them [19,20,21].

SCI severity scales have been proposed throughout to be able to estimate the functional prognosis of victims of these traumatic events. These tools can be divided into scales for assessing the neurological deficit due to trauma, and functional scales that assess the ability to perform daily activities, self-care, etc.

### 3.1. Neurological Deficit Assessment Scales

Table 1 is the neurological deficit assessment scales.

### 3.2. Scales for Functional Assessment of Spinal Cord Injuries

Table 2 is scales for functional assessment of spinal cord injuries. These instruments are used specifically by specialists in the field of medical and neuromotor recovery, being represented by: the Barthel Index (BI), Modified Barthel Index (MBI), Functional Independence Measure (FIM), Quadriplegic Index of Function (QIF), Spinal Cord Independence Measure (SCIM), Walking Index for Spinal Cord Injury (WISCI), and Spinal Cord Injury Functional Ambulation Inventory (SCI-FAI). Scales of functional assessment of spinal cord injuries determine a person’s ability to perform their activities of daily living (ADL), thus determining the ability of an individual to self-care, walk alone, etc. These clinical tools are usable for a wide range of neurological conditions, especially for VMT lesions: QIF, SCIM, and SCI-FAI. Of these scales, the Barthel Index is the most widely used.

### 3.3. Scales That Evaluate Both the Neurological Deficit and Functional Assessment of Patients with SCI

Table 3 is scales that evaluate both the neurological deficit and functional assessment of patients with SCI.

## 4. Conclusions

The closest scale to the need for standardization and the most accurate assessment of neurological deficits secondary to SCI is ASIA/IMSOP, adopted as the international standard for the neurological assessment of spinal cord trauma patients. The WISCI is a more accurate tool than the FIM for documenting changes in walking levels, but the FIM is more reliable in measuring patient self-care and independence.

## Figures and Tables

**Table 1:** Neurological deficit assessment scales.

Nr.	Scale	Year	When to Use	Advantage	Limitation	References
1.	Scala Frankel	1969	- Neurological deficit: A–E - A (complete neurological damage) - E (clinically normal)	- The first publicly available neurological scale - Synthetic - Clinically easy to use	- Unclear differentiation between grade C and D - Subjective nature in judging “usefulness” of any remaining motor movements - The level of the injury is not incorporated in the classification - Limited responsiveness to subtle neurological improvements during recovery.	[22,23,24,25]
2.	Bracken Scale	1978	- 2 subscales: 7 sensitive items and 5 motor items	- Acute hospitalization	- Clinically rarely used	[26,27]
3.	Lucas and Ducker’s Neuro-trauma Motor Index	1979	- Evaluates 23 muscles	- Predictive value of patient’s functional independence	- Heavy calculations in the evaluation of motor function	[28,29]
4.	Yale Scale	1981	- Tests sensory and motor function	- Numerical grading of selected functions below the level of the lesion	- The multiple calculations make it difficult to use in current practice	[29,30]
5.	Sunnybrook Scale	1982	- 10 degrees with motor and sensory deficit	-Differentiation of sensory and motor deficits between the equivalent degrees C and D from Frankel scale	- Multiple calculations	[31,32,33]
6.	American Spinal Injury Association (ASIA)	1984	- Derived from the Frankel scale - Designated as an assessment tool to classify baseline neurological impairment.	- Classifies neurological injuries based on a practical way to admission - Provides information regarding the improvement of the patient’s condition during the follow-up	- Does not reveal the objective anatomic origin of the causal injury - Does not decide injury severity	[34,35,36,37]
7.	Botsford Scale	1992	- Assesses motor and sensory function, rectal tone and bladder control - The motor score is obtained by testing 15 key muscles scored between 0–5 points (maximum 75 points) - The maximum sensitive score is 10 points - Anal tonus 10 points - Bladder tonus 5 points	- Introduces anal sphincter and bladder tonus control testing into the neurological assessment, as a measure of outstanding functionality - Can be used at the patient’s bedside - It does not require special tests other than those performed in a routine clinical neurological examination - Motor function is assessed in a functional assessment system	- Few clinical studies have used this scale.	[38,39]

**Table 2:** Functional evaluation scales of SCI.

Nr.	Scale	Year	When to Use	Advantages	Limitation	References
1	Barthel Index (BI)	1965	- Functional evaluation of patients with stroke and TVM - Score between 0–100 points, evaluates the tone of the anal sphincter, bladder tonus - Personal hygiene - Using the toilet - Food - Transfer from bed to cart and vice-versa - Mobility - Dressing - Climbing steps - Bathing	- Evaluates daily activities and some physiological functions - Easy to use	The examination time is long	[40]
2	Modified Barthel Index (MBI)	1989	- Allows anyone to assess the activities of daily living	- Measures independence in ADL	Evaluates only stroke patients	[41,42]
3	Functional Independence Measure (FIM)	1987	- Consists of 13 motor and 5 cognitive items with a score between 18 and 126 points - Divided into main categories and subcategories: self-care (feeding, brushing, bathing, dressing, toileting) sphincter control (bladder and anal), transfer mobility (transfer from bed to chair or wheelchair, from toilet to wheelchair, using the shower), locomotion (walking/wheelchair, stairs), communication (understanding, expression), social cognition (social interaction, problem solving, memory)	- Measures global independence during specific functional tasks - Records progress results - Specifies the patient’s functional mobility and independence - More sensitive, detailed and comprehensive compared to the Barthel index, socio-economically meaningful improvements	Long examination time	[43,44,45]
4	Quadriplegia Index of Function QIF	1980	- Quadriplegic patients	- More sensitive and reliable than the Barthel Index	Use only in quadriplegic patients	[46,47]
5	Spinal Cord Independence Measure (SCIM)	1997	- Functional categories: selfcare (subscore 0–20), breathing and sphincter management (0–40) and mobility (0–40); - Final score between 0 and 100	- Disability scale developed specifically for patients with spinal cord injuries, to make functional assessments sensitive to changes occurring in the follow-up of patients with para- or tetraplegia	Use only in SCI	[48,49,50]
6	Walking Index for Spinal Cord Injury WISCI	2000	- Originally described with 19 levels, it was revised and expanded in 2001 to include 21 levels, thus resulting in the WISCI II for use in clinical trials	- Measures improvement in walking after SCI	Cannot be used in clinical trials	[51]
7	Walking Index for Spinal Cord Injury II (WISCI II)	2001	- Improvements in walking following SCI	- Incorporates physical assistance, the use of dental aids and appliances to be able to adapt to the needs of patients with varying degrees of post SCI impairment - Self-explanatory	Does not take psycho-metric properties into account	[52,53]
8	Spinal Cord Injury Functional Ambulation Inventory (SCI-FAI)	2001	- 6 parameters: weight shift, step width, step rhythm, step height, foot contact and step length - Each limb is scored individually, so the same score for each limb indicates symmetry between bilateral limbs, the patient is assessed from the frontal plane, then sagittal - Divided into 3 areas: walking (6 parameters and symmetry between the lower limbs)—maximum score of 20 points, 14 points for the use of assistive devices and 5 points for the walking mobility score	- Observational assessment of walking ability in people with SCI - Can be performed directly or from video recordings	- The three scores of the SCI-FAI instrument are intended to measure different domains of functioning - Not relevant to combine them to obtain a global score	[54,55]

**Table 3:** Scale for the evaluation of the neurological deficit and functional assessment of patients with SCI.

Nr.	Scale	Year	When to Use	Advantages	Limitation	References
1.	American Spinal Injury Association/International Medical Society of Paraplegia Standards (ASIA/IMSOP)	1992	- Assesses sensory level, motor function - Incorporated in the Functional Independence Measure (FIM)	- Functional status based on their ability to perform ADL plus social interaction - Good discrimination in severity of SCI - Predictability of outcome	- Weak interobserver reliability for the grading of incomplete SCI	[56]

## Data Availability

Not applicable.
